# A Prevalence Anatomic-Imaging Study of the Posterior Inferior Cerebellar Artery’s Origin

**DOI:** 10.3390/medicina60091397

**Published:** 2024-08-26

**Authors:** Ana-Maria Davidoiu, Mugurel Constantin Rusu, Corneliu Toader, Petrinel Mugurel Rădoi

**Affiliations:** 1 Doctoral School, Faculty of Medicine, “Victor Babeş” University of Medicine and Pharmacy, RO-300041 Timişoara, Romania; ana-maria.davidoiu@umft.ro; 2 Department 1, Division of Anatomy, Faculty of Dentistry, “Carol Davila” University of Medicine and Pharmacy, RO-020021 Bucharest, Romania; 3 Department 6–Clinical Neurosciences, Division of Neurosurgery, Faculty of Medicine, “Carol Davila” University of Medicine and Pharmacy, RO-020021 Bucharest, Romania; corneliu.toader@umfcd.ro (C.T.); petrinel.radoi@umfcd.ro (P.M.R.); 4 Clinic of Neurosurgery, “Dr. Bagdasar-Arseni” Emergency Clinical Hospital, RO-041915 Bucharest, Romania

**Keywords:** cerebellum, vertebral artery, cerebellar artery, foramen magnum, PICA

## Abstract

*Background and Objectives*: Typically, the vertebral arteries (VAs) enter the posterior fossa through dural rings and further unite, forming the basilar artery. The posterior inferior cerebellar artery (PICA) is usually a branch of the V4 segment of the VA (intradural origin). It may also leave the V3 suboccipital segment of the VA (extradural origin). The transdural origin of the PICA within the VA’s dural ring has been consistently overlooked. A study was designed to determine the topographical patterns of the PICA’s origin. *Materials and Methods*: Determinations were performed in a retrospective sample of 225 computed tomography angiograms. Four types of PICA origin were documented: type 0, absent PICA; type 1, the extradural origin of the PICA from the V3 segment of the VA; type 2, the transdural origin of the PICA within the dural ring; and type 3, the intradural origin of the PICA from the V4 segment of the VA. The bilateral symmetry of types was also investigated. *Results*: Out of 450 VAs, type 0 (absent PICA) was found in 36%, type 1 (extradural) in 0.44%, type 2 (transdural) in 5.56%, and typical type 3 in just 58%. In types 1 and 2, the PICA entered the posterior fossa through the dural ring and the marginal sinus. In the overall group (N = 225), the type combinations 1_1, 1_2 and 1_3 were not found. Bilaterally absent PICAs occurred in 18.67%. The bilateral combinations 0_1/0_2/0_3/2_2/2_3/3_3 were found, respectively, in 0.89%/3.11%/30.67%/1.78%/4.44%/40.44%. Four of the seventy-eight PICAs opposite to an absent one, three intradural and one transdural, were true bihemispheric PICAs. *Conclusions*: The PICAs with extradural or transdural origins are facultative contents of the dural ring and are at risk during neurosurgical approaches in the foramen magnum. Rare bihemispheric PICAs could originate either intradurally or within the dural ring.

## 1. Introduction

Typically, the vertebral artery (VA) enters the posterior fossa through a funnel-shaped dural ring, joins the opposite one, and forms the basilar artery (BA) [1,2]. The vertebrobasilar system supplies the cerebellar arteries. The posterior inferior cerebellar artery (PICA) is typically the most distal branch of the intradural V4 segment of the VA [3,4]. It may also arise from the BA [5]. It vascularises the postero-inferior part of the cerebellum [5]. The medial branch of the PICA supplies a dorsomedial cerebellar territory that includes the dorsolateral portion of the medulla oblongata, and the lateral branch of the PICA supplies an anterolateral cerebellar area. Still, it never supplies the medulla oblongata [6]. The PICA has a highly variable origin; it usually arises from the VA about 14–17 mm proximal to the origin of the BA [4,7]. It may be double; the VA may terminate as the PICA, have an extradural origin, or be absent [5,7,8].

PICA aneurysms usually arise from the VA-PICA junction and the proximal segment of the PICA [9] but can also occur distally on the intradural PICA or at the dural sac penetration, accounting for 0.5–3% of all cerebral aneurysms [10]. Extracranial vascular pathology rarely leads to intracranial subarachnoid haemorrhage; possible etiologic lesions include aneurysms of an extradural PICA [10]. 

Different investigations of PICA have indicated either its extradural or intradural origin without distinguishing the origin of PICA from the VA at the dural sac crossing [9,10,11,12,13,14,15,16,17,18,19,20,21,22,23,24,25]. A PICA of extradural origin may originate from the horizontal portion of the VA just outside the dura mater, laterally above the transverse foramen of the atlas, or from the vertical portion of the V3 segment of the VA [26]. When originating from the vertical portion of the VA, the PICA penetrates the dura mater between C2 and C1 [26].

We decided to perform a prevalence study of the origin of PICA. We decided on an angioCT study because most studies that have evaluated anatomic variations in the suboccipital segment of VA have used this imaging modality [27]. 

## 2. Materials and Methods

Determinations were performed in a retrospective sample of 225 adult cases, 127 men and 98 women. Exclusion criteria were as follows: inadequate scans to observe the anatomy of the VA and BA, cervical, suboccipital, and intracranial pathologic processes distorting their anatomical features, previous surgery in the craniocervical region and/or the posterior cranial fossa, and hyperextension or excessive lateral rotation of the neck during CT scan. No cases were excluded. The research followed the principles of the World Medical Association Code of Ethics (Declaration of Helsinki). The Ethics Committee of the “Dr. Bagdasar-Arseni” Emergency Clinical Hospital approved the study (approval no. 2093/1 March 2022).

The CT angiograms were performed with a 32-slice scanner (Siemens Multislice Perspective Scanner, Erlangen, Germany), with a 0.6 mm collimation and a reconstruction of 0.75 mm thickness with 50% overlap for a multiplanar maximum intensity projection and three-dimensional volume rendering technique, as described previously [28]. The cases were documented using the Horos 3.3.6 software for iOS (Horos Project, Annapolis, MD, USA). Evaluations of the presence and types of PICAs were independently performed by an experienced anatomist (author #2) and two neurosurgeons (authors #3 and #4). The positive results were identical and were validated by each author.

We defined and determined four types of PICA origin: type 0, absent PICA; type 1, the extradural (low) origin of the PICA from the V3 suboccipital segment of the VA; type 2, the transdural origin of the PICA at the passage of the VA through the dural ring; and type 3, the intradural origin of the PICA from the V4 segment of the VA. The bilateral symmetry of types was also investigated.

## 3. Results

In the overall group (N = 225), on the right side, we found 85 absent PICAs (type 0, 37.78%) and 11 type 2 (transdural, 4.89%) PICAs. The typical intradural (type 3) origin of PICAs was recorded in 129 cases (57.33%). No type 1 (extradural) PICAs were recorded on the right side. On the left, type 0 PICA (absent) was recorded in 77/225 cases (34.22%), type 1 PICA with extradural origin just outside the dural ring was evidenced in 2/225 cases (0.89%), type 2 PICA with transdural origin presented in 14/225 cases (6.22%), and type 3 PICA with intradural origin was recorded in 132/225 cases (58.67%). 

In males (N_M_ = 127), on the right, PICA type 0 (absent) was recorded in 46 cases (36.22%), extradural type 1 PICA origin was not found, type 2 (transdural origin of PICA) was evidenced in 10 cases (7.87%), and intradural origin of PICA (type 3) was evidenced in 71 cases (55.91%). On the left side, in males (N_M_ = 127), PICA type 0 was recorded in 43 cases (33.86%), there were no extradural origins of PICA (type 1), transdural origin (type 2) was found in 8 cases (6.3%), and intradural origin of PICA (type 3) was found in 76 cases (59.84%).

In females (N_F_ = 98), on the right, PICA type 0 (absent) was recorded in 39 cases (39.8%), extradural type 1 of PICA origin was not found, type 2 (transdural origin of PICA) was evidenced in one case (1.02%), and intradural origin of PICA (type 3) was evidenced in 58 cases (59.18%). On the left side, in females (N_F_ = 98), PICA type 0 was recorded in 34 cases (34.69%), PICA type 1 (extradural) was recorded in 2 cases (2.04%), transdural origin (type 2) was evidenced in 6 cases (6.12%), and intradural origin of PICA (type 3) was evidenced in 56 cases (57.14%).

In the overall group of 450 left and right sides, PICA was missing (type 0) in 162 instances (36%), extradural type 1 was identified for 2 PICAs (0.44%), transdural type 2 was identified for 25 PICAs (5.56%), and intradural localisation of PICA’s origin (type 3) was detected in 261 arteries (58%). In the male sublot (N_M_ = 254 sides), PICA type 0 was identified 89 times (35.04%), type 1 was not detected, type 2 (transdural) was detected 18 times (7.09%), and type 3 (intradural) was detected 147 times (57.87%). In the female sublot (N_F_ = 196 sides), PICA was missing in 73 instances (37.24%), extradural type 1 PICA was detected 2 times (1.02%), transdural type 2 PICA was present 7 times (3.57%), and intradural type 3 PICA was detected 114 times (58.16%).

Concerning the bilateral combinations of PICA origin types, in the overall group (N = 225), we did not identify the combinations 1_1, 1_2, and 1_3; the combination 0_0 (bilaterally absent PICA) was identified in 18.67%, the combination 0_1 (absent PICA and transdural PICA) in 0.89%, the combination 0_2 (absent PICA with transdural PICA) in 3.11%, the combination 0_3 (unilateral intradural PICA) in 30.67%, the combination 2_2 (bilateral transdural PICA) in 1.78%, the combination 2_3 (transdural PICA with intradural PICA) in 4.44%, and the combination 3_3 (bilateral intradural PICA) in 40.44%.

**Figure 1:**
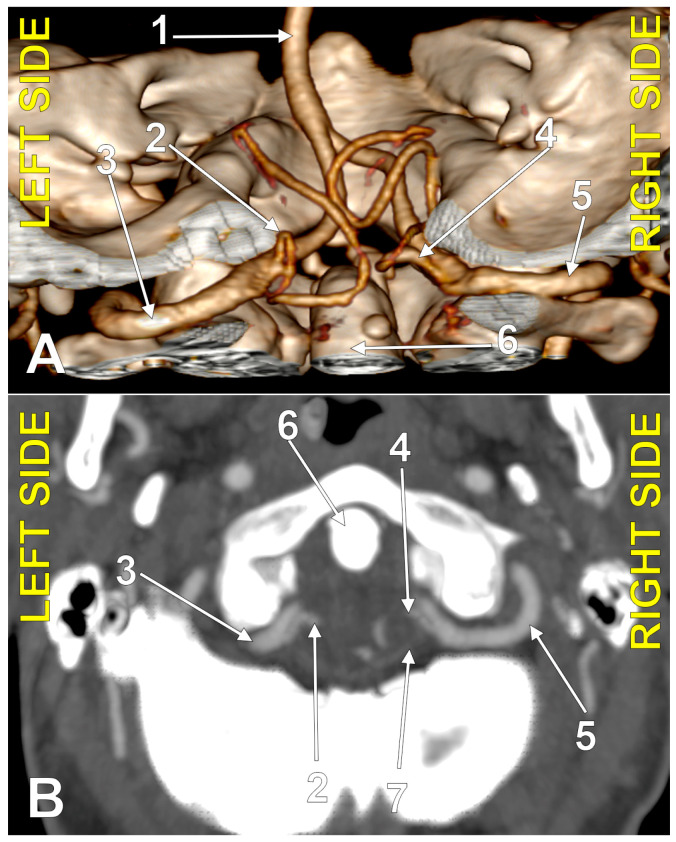
Bilaterally symmetrical posterior inferior cerebellar arteries of transdural (type 2) origin (combination 2_2). (**A**): Three-dimensional volume rendering, postero-inferior view. (**B**): Axial slice, superior view; 1. basilar artery; 2. left posterior inferior cerebellar artery; 3. left vertebral artery; 4. right posterior inferior cerebellar artery; 5. right vertebral artery; 6. dens of axis; 7. dural ring.

**Figure 2:**
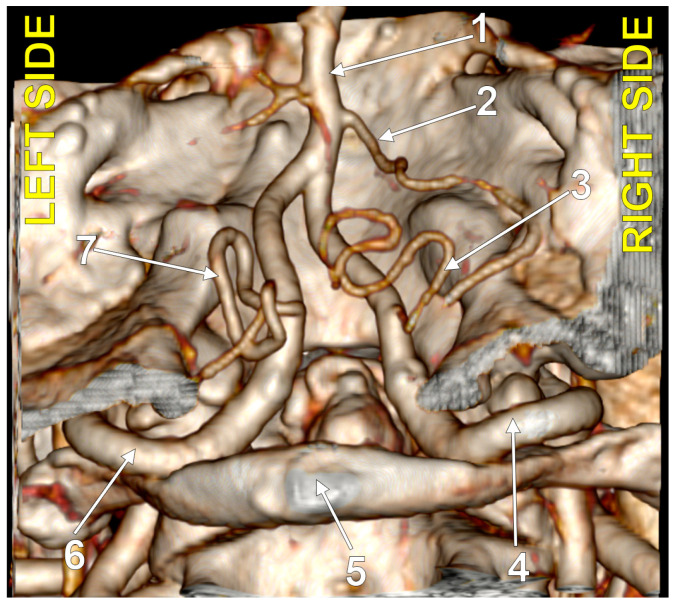
Bilaterally symmetrical posterior inferior cerebellar arteries with intradural (type 3) origin (combination 3_3). Three-dimensional volume rendering, postero-inferior view. Numbers as follows: 1. basilar artery; 2. anterior inferior cerebellar artery; 3. right posterior inferior cerebellar artery; 4. right vertebral artery; 5. posterior arch of the atlas; 6. left vertebral artery; 7. left posterior inferior cerebellar artery.

In men (N_M_ = 127), there were no bilateral combinations that included type 1 PICA (extradural origin); combination 0_0 was detected in 18.9% (absent bilateral PICA), combination 0_2 (unilateral transdural PICA) in 3.15%, combination 0_3 (unilateral PICA with intradural origin) in 29.13%, combination 2_2 (bilateral transdural PICA) in 2.36% (Figure 1), combination 2_3 (transdural PICA and intradural PICA) in 6.3%, and combination 3_3 (bilateral intradural PICA) in 40.16% (Figure 2).

**Figure 3:**
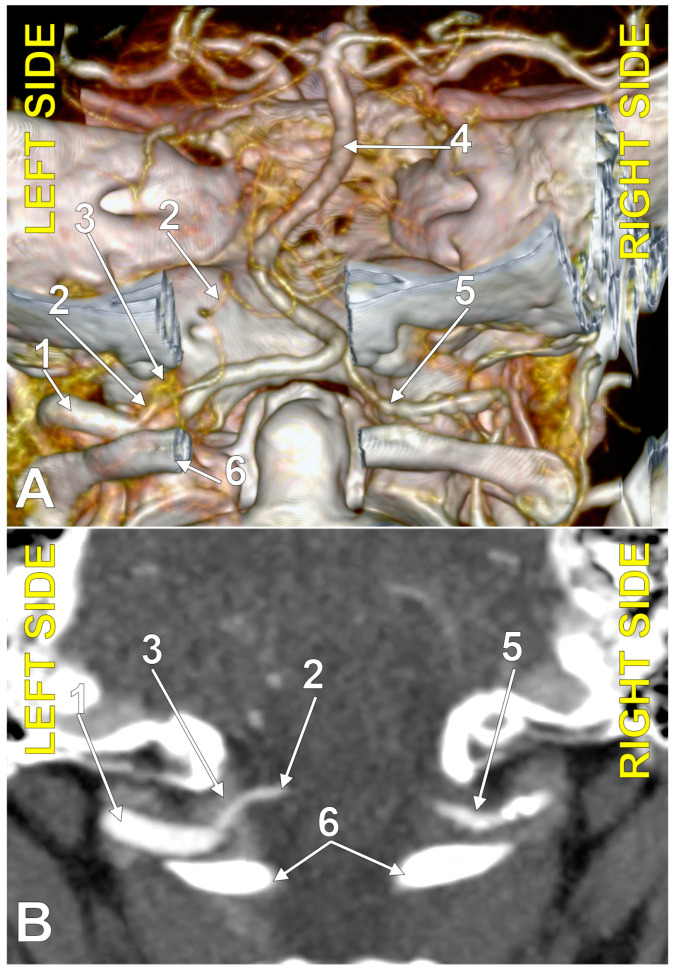
Posterior inferior cerebellar artery with extradural origin (type 1); bilateral asymmetry (combination 0_1). (**A**): Three-dimensional volume rendering, postero-inferior view. (**B**): Axial section, superior view. Numbers as follows: 1. left vertebral artery; 2. left posterior inferior cerebellar artery; 3. marginal sinus (plexus); 4. basilar artery; 5. right vertebral artery; 6. posterior arch of the atlas.

In females (N_F_ = 98), the combination 0_0 (bilaterally absent PICA) was identified in 18.37%, the combination 0_1 (unilateral PICA with immediate extradural origin) in 2.04% (Figure 3), the combination 0_2 (unilateral PICA with transdural origin) in 3.06%, and the combination 0_3 (unilateral PICA with intradural origin) in 32.65%. Bilateral combinations 1_1, 1_2, and 1_3 were not identified, combination 2_2 (bilateral transdural PICA with transdural origin) was detected in 1.02%, combination 2_3 (transdural + intradural) in 2.04%, and combination 3_3 (bilateral PICA with intradural origin) in 40.82%.

**Figure 4:**
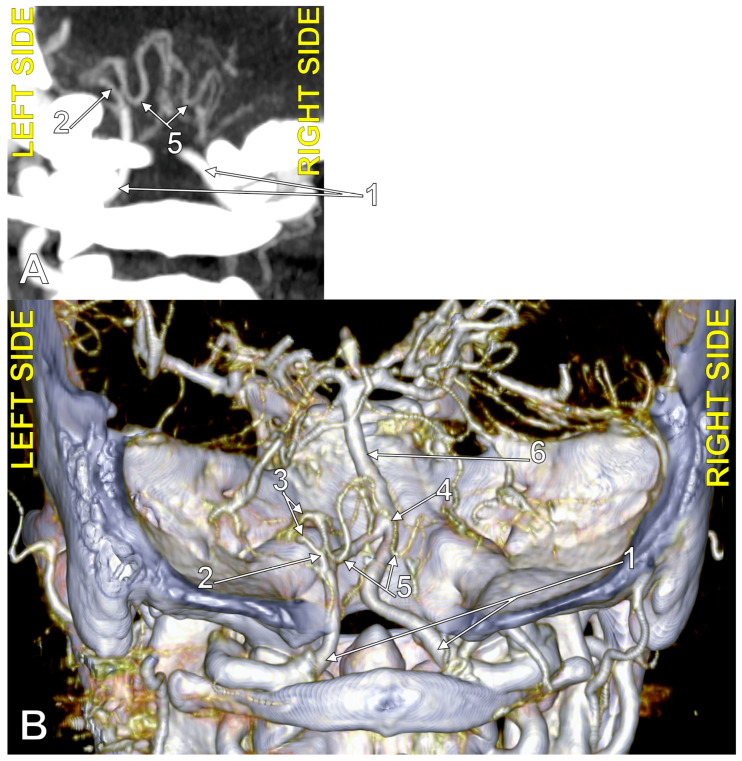
Bihemispheric type 3 posterior inferior cerebellar artery (BPICA). (**A**). Coronal section, posterior view. (**B**). Three-dimensional volume rendering, posterior view; 1. vertebral artery; 2. BPICA; 3. ipsilateral cerebellar hemispheric distribution; 4. contralateral cerebellar hemispheric distribution; 5. inferior loops of BPICA; 6. basilar artery.

**Figure 5:**
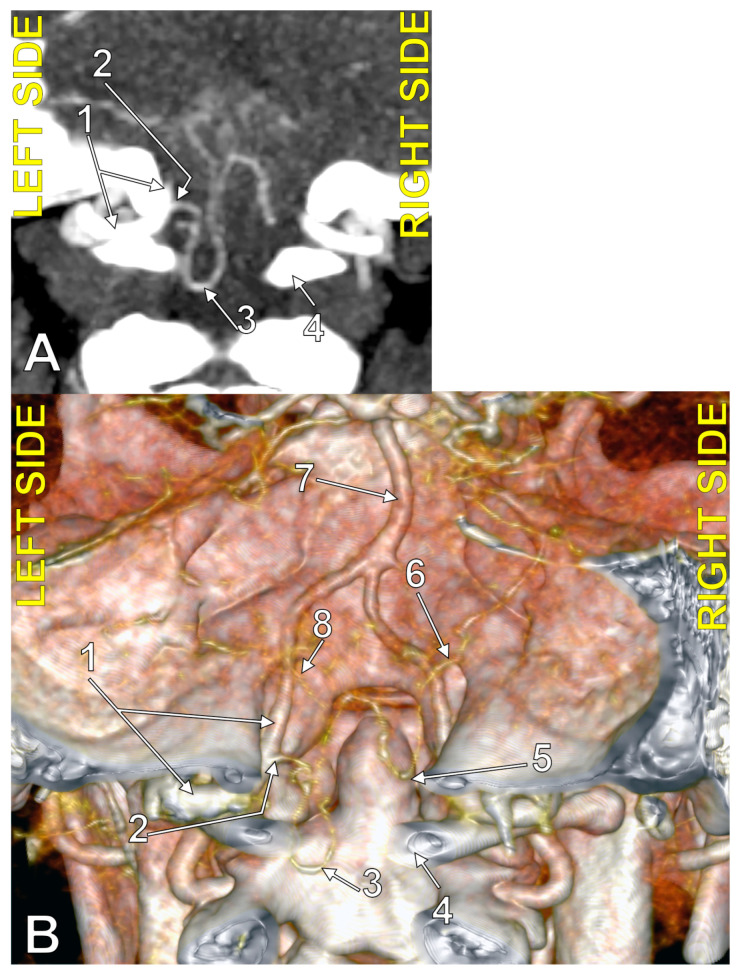
Bihemispheric type 2 posterior inferior cerebellar artery (BPICA). (**A**). Coronal section, posterior view. (**B**). Three-dimensional volume rendering, posterior view; 1. left vertebral artery; 2. origin of left BPICA; 3. left inferior loop of BPICA; 4. posterior arch of atlas; 5. inferior loop of right PICA; 6. right PICA proper; 7. basilar artery; 8. left PICA proper.

**Figure 6:**
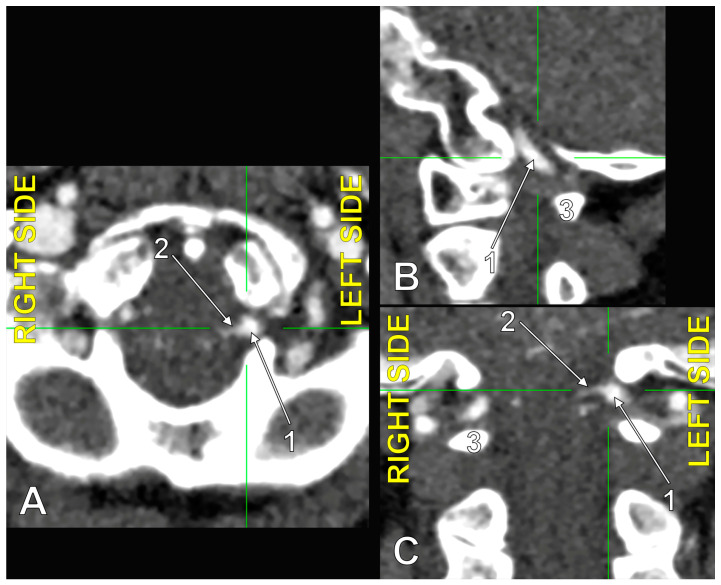
The origin of the left type 2 bihemispheric left posterior inferior cerebellar artery (BPICA). (**A**). Axial section, inferior view. (**B**). Sagittal section, lateral view. (**C**). Coronal section, anterior view; 1. left vertebral artery; 2. left BPICA; 3. posterior arch of the atlas.

In the overall group (N = 225), we had 78 bilateral combinations with absent PICA (type 0). Of the 78 PICAs contralateral to a type 0, 4 were true bihemispheric PICAs (BPICA, 5.15%). Compared to the overall group of 225 cases, this results in an incidence of 1.77% BPICAs. Of the four recorded BPICAs, three were 0_3 bilateral combinations (Figure 4), and one was a 0_2 bilateral combination (Figure 5). The latter showed a transdural type 2 origin of a left BPICA (Figure 6); from the origin, it descended 1.44 cm into the spinal dural sac, then described a first inferior loop 1.97 mm below the posterior arch of the atlas. It then ascended into the vertebral canal, reaching the foramen magnum and dividing posterior to the medullospinal junction into the two PICAs, left and right. That right PICA had an initially ascending trajectory, followed by an upper loop at the level of the superior contour of the foramen magnum, and then descended to realise the inferior loop at the level of the respective atlantooccipital joint, 3.88 mm medial to the right VA. Thus, that BPICA appeared as a common trunk originating from the left VA of the two PICAs, left and right. The inferior loop of the BPICA corresponded to the inferior loop of the left PICA, whereas the respective right PICA described its inferior loop.

## 4. Discussion

This study is the first one indicating that the PICA could originate within the dural ring, not just from an intra- or extradural site of origin. The prevalences of three anatomical possibilities of the origin of PICA were established. The course of the PICA through the dural ring was overlooked in previous studies. We demonstrated that the exact site of origin of the PICA can be adequately determined on correlated two-dimensional slices and verified on three-dimensional volume renderings. The results of this study demonstrate that the typical anatomy of the PICA is not so frequent, and variations such as the transdural origin, absence, or BPICA can occur. The postero-lateral approach of the foramen magnum equally targets extradural and intradural structures [29]. Care should be taken with type 1 or 2 of the PICA to avoid an unwanted haemorrhage or a cerebellar infarction. A BPICA should be carefully managed, and its transdural origin increases the risk of surgical lesions if they are not identified preoperatively. Such rare anatomical variations of PICA may escape the attention of neuroradiologists and neurosurgeons, as Yasargil commented [30]. 

### 4.1. Typical PICA

The PICA is exposed in dealing with neoplasms located from the cerebellopontine angle to the craniocervical junction, aneurysms arising at the PICA origin, arterial dissections at the PICA-VA junction, arteriovenous malformations, posterior fossa ischemia requiring bypass, anomalies at the craniocervical junction, and dysfunction of the lower cranial nerves [5]. Akgun et al. (2013) found typical vertebrobasilar anatomy in only 34.8% [31]; here, we found typical PICA anatomy (type 3) in 58% of VAs. Anatomical variations in the PICA’s origin are a significant risk factor, especially in the posterior approach to the V3 segment of the VA [27].

### 4.2. Absent PICA

The PICA is absent in 2–26% of cases [32]. Our study found that the PICA (450 VAs) was absent in 36% of cases, a prevalence above the previously known maximum limit. Absent PICA on the right was recorded by Akgun et al. (2013) in 17.8% of cases and on the left in 11.1% [31]. In the lot investigated by the present study, PICA was absent in 37.78% on the right and 34.22% on the left, a higher prevalence of PICA absence than in the respective research studies.

A bilaterally absent PICA was identified in 1/50 cases, or 2% [7]. In the present study, bilateral absent PICA had a prevalence of 18.67%, which is obviously much higher. Furthermore, in a study using dissection and latex injection on 25 cadavers, Lister et al. (1982) found neither bilateral absence of PICA nor VAs terminated as PICAs [17]. Salamon and Huang (1976), cited by Lister et al. (1982), found the unilateral absence of PICA in 26% and the bilateral absence of PICA in only 2% of cases [33]. Margolis and Newton (1974), cited by Lister et al. (1982), reported the absence of PICA in 15% of cases [34]. Macchi et al. (2003) documented that PICA may be unilaterally absent in 15–26% and bilaterally absent in 2–2.5% of cases [35]. These authors found no bilateral absence of PICA in 40 anatomic specimens but found its unilateral absence in 2.5% of specimens [35]. 

Operative approaches to the posterior fossa could be optimised by understanding the three neurovascular complexes defined by Rhoton Jr. (2000): the upper complex related to the superior cerebellar artery, the middle complex associated with the anterior inferior cerebellar artery, and the lower complex related to the PICA [5]. The upper complex includes the superior cerebellar artery, midbrain, cerebellomesencephalic fissure, superior cerebellar peduncle, tentorial surface of the cerebellum, and the third, fourth, and fifth cranial nerves [5]. The middle complex includes the anterior inferior cerebellar artery (AICA), pons, middle cerebellar peduncle, cerebellopontine fissure, petrosal surface of the cerebellum, and the sixth, seventh, and eighth cranial nerves [5]. The lower complex includes the PICA, medulla, inferior cerebellar peduncle, cerebellomedullary fissure, suboccipital surface of the cerebellum, and the ninth, tenth, eleventh, and twelfth cranial nerves [5]. However, the typical neurovascular complexes of Rhoton are modified by different variations of the cerebellar arteries. Less than 12% of cases have all the cerebellar arteries without anatomical variations [8]. Any of the three typical cerebellar arteries may be absent [5,36,37,38].

Anastomoses between the cortical segments of the cerebellar arteries, superior, AICA, and PICA, consisting of multiple hemispheric and some vermian branches, provide an alternative supply [39]. Three possibilities were distinguished: cerebellar hemispheres with the AICA dominating the PICA (type I), cerebellar hemispheres with the PICA dominating the AICA (type III), or cerebellar hemispheres with AICA and PICA of comparable size (type II) [40].

In isolated absent PICAs, the most common variation of the posterior circulation, irrigation to the absent PICA territory is provided from the ipsilateral AICA or the contralateral PICA via interarterial anastomosis [41]. In such cases, an occlusive process affecting the PICA also leads to ischemia and symptoms in the absent PICA’s territory [41].

### 4.3. Extradural Origin of the PICA

According to Stoodley et al. (2000), an extradural origin of the PICA was first described by Margolis and Newton (1974) [34,42]. When the PICA originates extradurally, its origin site is usually within 1 cm of the site at which the VA penetrates the dura [42]. Such an extradural PICA does not possess extradural branches, remains lateral and posterior to the brainstem, and does not supply the anterior brainstem [42]. 

Different authors have reported an extradural origin of the PICA between C1 and C2 [14,21,22,25]; we did not record this variant in the present study. Xu et al. (2018) found a 1% incidence of this variant of the extradural PICA [25]. Kim et al. (2016) found PICAs with extradural origins at C1 and C2 [16]. Of 346 PICAs, Brinjikji et al. (2009) found none originating below the posterior arch of the atlas [43]. Although numerous authors recorded the origin of the PICA from the V3 segment of the VA, they did not distinguish between an extradural and a transdural origin [9,11,13,14,15,16,17,18,19,20,21,22,23,24,25,30,44,45,46]. 

A recent systematic review documented that an extradural origin of the PICA was found in 175 out of 10,820 cases (1.6%) [27]. In 98 computed tomograms, Duan et al. (2010) did not record any extradural origin of the PICA [44]. O’Donnell et al. (2014) found this variant, the type 1 PICA that we recorded in 0.44% of 450 VAs here, in 4/975 cases (0.41%). Arslan et al. (2019) studied 200 computed tomograms and found extradural PICAs in just 1% [11]. However, higher incidences of extradural PICA resulted in other studies. Vaněk et al. (2017) studied the CT angiograms of 511 cases and found unilateral extradural PICAs in 4.1% [45]. Isaji et al. (2018) found that the PICA originated from the V3 segment in 9.5% of 284 sides, more frequently observed in the non-dominant VA than in the dominant VA [15]. Pekcevik and Pekcevik (2014) found an extradural origin of the PICA in 71/341 (20.8%) patients [8].

PICAs with extradural origins are exposed to injury during posterior approaches to the lower brainstem and upper cervical spine [46]. Pekcevik and Pekcevik (2014) discuss that knowing and reporting this variation is clinically significant because “it might be the origin of the subarachnoid haemorrhage due to associated aneurysm” [8]. However, an extradural aneurysm—certainly at the PICA’s origin—cannot bleed into the subarachnoid space. Only aneurysms of the intradural PICA can do so. Extradural exposure of the V3 segment of the VA risks injury of a PICA with an extradural origin if this variant is not identified preoperatively [30]. Diaz Day added comments to a report of extradural PICAs [30]; he discussed that the small size of an extradural PICA makes it vulnerable to being mistaken for a large posterior meningeal branch that emerged from the VA before it entered the dural ring, or for a posterior spinal artery. In these regards, Diaz Day suggested that it is best to practice strict preservation of any arterial branches from the VA just before its dural entrance until these can be tracked to their intradural course [30]. This would avoid interrupting a vessel that supplies the brain stem and cerebellum [30]. 

### 4.4. Transdural Origin of the PICA

Ogasawara et al. (2017) discussed that the anatomy of the PICA is highly variable, with PICA agenesis/hypoplasia, double/duplicated origins, and extracranial or epidural origins [47]. No mention is made of the possibility of PICA origin at the crossing of the dural ring of the VA.

Lister et al. (1982) studied specimens from just 25 cadavers; they classified the origin of the PICA as related to the foramen magnum and found 7/42 PICAs (0.16%) arising from the VAs below the level of that foramen [17]. The relation of those seven PICAs with the dural ring was not detailed. In 450 VAs, we found 2 type 1 PICAs (0.44%) and 25 type 2 PICAs (5.56%), which makes a 6% prevalence of the PICA originating beneath the level of the foramen magnum. This possibility is significant because if a PICA branches from the VA below the foramen magnum, vertebrobasilar insufficiency may occur due to pressure on the bloodstream from the edge of the foramen magnum [48].

The preoperative identification of an extradural PICA is essential in planning the surgical strategy to avoid intraoperative complications near the foramen magnum [30]. Equally important is the identification of a PICA of transdural origin.

### 4.5. The PICA and the Marginal Sinus

When the VA crosses the dural ring, it will also cross the marginal sinus, as we have shown in this study. Reports on the marginal sinus have been few, and most standard anatomy texts mention only that it is found near the foramen magnum [49]. Tubbs (2020) discusses that surgical access into the deep subdural space of the foramen magnum involves crossing the marginal sinus, as in decompressions for Chiari I malformations or posterior fossa tumours [49]. Neither Tubbs (2020) nor other authors who have published information on the anatomy of the marginal sinus [50] refer to the possible relationship of this sinus to a PICA originating below the level of the foramen magnum. The present study makes an anatomical contribution by demonstrating that a PICA of type 1 or 2 crosses the marginal sinus.

### 4.6. The PICA and the Dural Ring of the VA

The funnel-shaped dural ring of the VA also embeds the anterior spinal artery, the dentate ligament, the first cervical nerve, and the spinal root of the accessory nerve [1,51]. A PICA originating from the V3 segment of the VA is not usually listed as a facultative content of the dural ring [1,51]. A PICA with a transdural origin will be at especially high risk during the surgical opening of the dural sac. Fine et al. (1999) described that the extradural PICA, VA, and the first cervical nerve roots pass through the dura together, and the PICA further reaches between the dentate ligament ventrally and the spinal root of the accessory nerve dorsally [32]. The VA is surrounded by a periosteal sheath that invaginates into the dural ring, creating a double furrow for 3–4 mm [29]. The periosteal sheath is in continuity with the outer layer of the dura, and the VA is attached to the periosteal sheath [29].

A tumour localised to the dural ring will also compress a type 1 or 2 PICA; the other cerebellar arteries may compensate for the functional impairment. But PICA occlusion, whether traumatic or surgical, may also cause infarcts in the brainstem and cerebellum [3]. 

Patients with cerebellar infarcts have common vascular risk factors: hypertension, history of stroke, ischemic heart disease, and atrial fibrillation [6]. Arterial occlusion in such cases is located in the VA in 50% of cases, in the BA in 25% of cases, and in a cerebellar artery in 25% of cases [6]. Nearly all occlusions of the PICA, but only slightly more than half of occlusions of the VA, result in medullary or cerebellar infarction [5]. The true incidence of cerebellar infarctions seems to be unknown [52]. A total 3000 CT scans were evaluated, and 21 cases of cerebellar infarcts were identified [52]. Of these, ten (0.33%) were in the PICA’s territory [52]. The leading causes of PICA infarcts are extracranial large artery disease in 41% of cases, cardioembolism in 20% of cases, and in situ branch lesions in just 20% of cases [53].

Here, we found 6% extradural and transdural types of PICA. These are prone to compression in the dural ring of the VA before dividing into the terminal medial and lateral branches. Wallenberg’s lateral medullary syndrome could occur as the medullary branches emerge distally to the dural ring [46]. This could also be an unwanted result of the surgical dissection of the dural ring if neurosurgeons do not preoperatively identify a transdural course of the PICA or a variant anatomy of the VA at this level. If a VA injury occurs, rapid action is required to prevent exsanguination or catastrophic neurological damage [54].

### 4.7. The Bihemispheric PICA

In Bergman’s Encyclopedia of Human Anatomic Variations, the BPICA variant is not mentioned [36]. Cullen et al. (2005) reported four cases with this anatomic variant and discussed that its incidence is not known in the literature [55], although it is probably less than 0.1% (4/5000 cases) [56]. Rusu et al. (2013) reported a case of dissection in which the telovelotonsillar segment of a BPICA further gave off the two proper PICAs, each with a typical morphology [57]. Sardhara et al. (2013) reported a BPICA with low origin at the foramen magnum [58]. The present study also found such a low origin of the BPICA, with the artery originating from the VA at the level of its dural ring. Sardhara et al. (2013) did not localise BPICA to the dural ring. Carlson et al. (2013) reported eleven cases of BPICA: five were typical BPICAs with distal VA hypoplasia, two were BPICAs of vermian type, and one was atypical, with the PICA feeding a contralateral arteriovenous malformation [59]. Ogasawara et al. (2017) described two kinds of BPICA: true bihemispheric, distributed to the contralateral cerebellar hemisphere, and the vermian variant, with only medial distribution [47]. Imahori et al. (2021) published a neurosurgical technical note in which they included a case of BPICA that they indicated as a dominant PICA that also vascularised the contralateral cerebellar hemisphere [60]. Uchino (2022) presents a case with BPICA in his atlas of imaging anatomy [61]. Boggio et al. (2023) found a BPICA upon dissecting a cadaver with Chiari I malformation [62]. However, the evidence presented by these authors is not convincing for the bihemispheric distribution of the artery. BPICAs aneurysms were also reported [47,63,64,65].

The BPICA is a rare anatomic variant mostly presented in case reports. Although Cullen et al. (2005) estimated a <0.1% incidence of it, Carlson et al. (2013) reported 11 BPICA cases, of which 9 resulted from a batch of 250 angiograms, which leads to an incidence of 3.6% for the BPICA [56,59]. In the present study of 225 cases, we found an incidence of 1.77% of BPICA. Moreover, we demonstrated that the BPICA could have either an intradural or a transdural type of origin from the VA. To our knowledge, these different possibilities have not been reported previously.

### 4.8. The Caudal Loop of the PICA

Lateral C1-C2 puncture, used for cervical myelography, cerebrospinal fluid sampling, percutaneous cervical cordotomy, or cervical intrathecal catheter placement, may damage a PICA with an aberrant course [43]. We found an inferior or caudal loop of a BPICA reaching 1.97 mm below the posterior arch of the atlas. This is a scarce variation; Brinjikji et al. (2009) found caudal loops of the PICA reaching inferior to the posterior arch of the atlas in just 0.6% of cases [43]. These authors discussed that surgeons should scrutinise any available arterial imaging before planning C1-C2 puncture [43].

## 5. Conclusions

The PICAs with extradural or transdural origins are facultative contents of the dural ring and are at risk during neurosurgical approaches in the foramen magnum. Rarely, BPICAs could originate intradurally or within the dural ring, but they may project caudal loops lower than the posterior arch of the atlas.

## Data Availability

No new data were created or analysed in this study. Data sharing does not apply to this article.
